# Retrospective Clinical Outcomes of Keratoplasty Using Human Donor Corneas Preserved in Eusol-C Hypothermic Storage Medium

**DOI:** 10.3390/jcm13247606

**Published:** 2024-12-13

**Authors:** Rossella Anna Maria Colabelli Gisoldi, Giulio Pocobelli, Umberto Rodella, Laura Giurgola, Claudio Gatto, Gemma Lodato, Giacinta Buffon, Carlo Nucci, Jana D’Amato Tóthová, Augusto Pocobelli

**Affiliations:** 1 Ophthalmology Unit–Eye Bank of Rome, San Giovanni Addolorata Hospital, 00184 Rome, Italy; 2 Ophthalmology Unit, Department of Experimental Medicine, University of Rome Tor Vergata, 00133 Rome, Italy; 3 Research and Development, AL.CHI.MI.A. S.R.L., 35020 Ponte San Nicolò, Italy

**Keywords:** hypothermic storage, corneal transplantation, corneal preservation, keratoplasty, donor cornea, Eusol-C, visual acuity, corneal transparency, organ-culture, Tissue-C

## Abstract

**Objective:** To evaluate the clinical outcomes of cornea transplantation (penetrating keratoplasty, Descemet membrane endothelial keratoplasty, Descemet stripping automated endothelial keratoplasty, and deep anterior lamellar keratoplasty) using donor corneas stored in Eusol-C hypothermic storage medium compared to corneas stored in organ-culture. **Methods**: The clinical outcomes of 92 patients who underwent corneal transplantation with human donor corneas stored in Eusol-C medium at 2–8 °C were retrospectively evaluated. The control group consisted of 169 patients who received corneas organ-cultured at 31 °C. Donor age, sex, death-to-preservation time, and storage time were recorded. Endothelial cell (EC) density (ECD), EC mortality, and EC morphology scores were evaluated during storage in both groups. Complication rates, visual outcomes, and corneal transparency were monitored for up to six months. **Results**: The mean storage in Eusol-C time was 7.7 ± 2.5 days, while organ-culture time was 14.2 ± 4.0 days. In the Eusol-C group, ECD was 2398 ± 354 cells/mm^2^, with an average EC morphology score of 3.4 ± 0.7/4. Approximately 28% of the corneas in the Eusol-C group had no EC mortality. In the organ-culture group, ECD was 2256 ± 328 cells/mm^2^, with an average EC morphology score of 3.5 ± 0.5/4, and 42% were devoid of EC mortality. No complications, such as re-bubbling, were observed in both groups during surgery. Transparent corneas were achieved in 81.3% of the Eusol-C group the day after surgery. Mean corrected distance visual acuity (CDVA) at 3 and 6 months was 4.5 ± 4.0/10 and 5.4 ± 3.7/10 for the Eusol-C group and 5.0 ± 2.9/10 and 5.7 ± 2.8/10 for the organ-culture group, with no statistical differences observed between the groups. No graft failure was observed up to three months. Graft rejection occurred in the Eusol-C group and in the organ-culture group in, respectively, one and two cases at the six-month follow-up. **Conclusions**: Comparable surgical outcomes were achieved with donor corneas stored in both hypothermic Eusol-C and organ-culture media.

## 1. Introduction

Corneal transplantation is the most common transplant performed worldwide. It is a well-established surgical procedure for restoring vision and for treating various corneal pathologies [1].

The availability of donated corneas is a prerequisite for successful transplantation and is currently the major limiting factor for this surgery, with a significant worldwide waiting list [2]. Accessibility of corneal tissue for transplantation is a concern that is addressed through the activities of Eye Banks, which include the responsibilities of procurement, preservation, ensuring quality control, and preparation of tissues for surgical procedures.

Preservation methods play a crucial role in maintaining the viability and quality of donor corneas and ensuring favorable post-transplantation outcomes, and several techniques have been already described in this regard [3,4].

Cryopreservation has only been relegated to specific circumstances, favoring less complex and safer techniques. Organ culture (31–37 °C) is widely used in Europe and allows for extended storage periods of up to four weeks. During this time frame, strict microbiological controls are performed to prevent the risk of host infection [5,6]. Hypothermic storage (2–8 °C) is the most used storage method in the United States of America. It is a relatively more straightforward technique than organ culture and allows storage for up to 14 days without the need for microbiological checks during the storage period [5,6]. Eusol-C (AL.CHI.MI.A. S.R.L., Ponte San Nicolò, Italy) is a widely used medium for hypothermic storage of donor corneas. It provides a balanced solution that optimizes corneal hydration and supports endothelial cell viability and overall corneal quality. Eusol-C has an equal formulation to that of the Corneal Chamber (AL.CHI.MI.A. S.R.L., Ponte San Nicolò, Italy): the only difference between the two medical devices is the reference market (Eusol-C reference market: United States of America; Corneal Chamber reference market: Europe).

Most of the literature addressing Eusol-C has focused on the quality of the human cornea during preservation, demonstrating favorable maintenance of endothelial cell viability and overall corneal integrity up to 14 days [7,8,9,10,11,12]. Conversely, only a few studies have evaluated the clinical outcomes of corneal transplantation using human donor corneas stored in Eusol-C and its efficacy in clinical practice [13].

To evaluate the safety and the clinical outcomes of corneal transplantation using corneas stored in Eusol-C medium at 2–8 °C for up to 14 days at the Eye Bank of Rome, a retrospective analysis was performed on a cohort of 92 patients who underwent various types of corneal transplantation procedures (penetrating keratoplasty PK, deep anterior lamellar keratoplasty DALK, Descemet membrane endothelial keratoplasty DMEK, Descemet stripping automated endothelial keratoplasty DSAEK) at the San Giovanni Addolorata Hospital in Rome. As a control group, 169 patients treated with corneas stored in organ culture at 31 °C for up to 28 days at the same eye bank were included.

In particular, this study also evaluated the potential occurrence of surgical complications, including those related to tissue preparation, corneal transparency, and visual acuity, within six months after corneal transplantation.

## 2. Materials and Methods

### 2.1. Corneal Recovery and Storage

A retrospective analysis of the clinical outcomes of a total of 261 patients who underwent corneal transplantation at the San Giovanni Addolorata Hospital in Rome between January 2020 and July 2022 using human donor corneas stored at the Eye Bank of Rome was performed. This retrospective study was conducted according to the guidelines of the Declaration of Helsinki and approved by the Central Ethics Committee of Lazio, Italy (protocol number 0022/2024; Rif. 7468; date of approval: 10 January 2024).

The procurement followed the guidelines of the Italian National Transplant Center, which defines the eligibility requirements of the potential donor; the age range accepted for donation is 3–80 years. The pathological and social history of the potential donor were collected, including physical inspection, macroscopic evaluation of the tissues, evaluation of the death report by the anatomopathological or forensic pathologist, and an interview with the family.

The corneal tissues were harvested within 24 h from donor decease. Demographic data (donor age and sex) and time from death to preservation (i.e., the time from donor decease to cornea procurement) were also recorded. Corneal tissues were collected within 24 h of donor death. Procurement was performed under sterile conditions by appropriately trained ophthalmologists according to the European Eye Bank Association (EEBA) guidelines [14]. All retrieved corneas underwent a microbiological evaluation at every change of medium.

In the study group, 92 human donor corneas were stored in Eusol-C storage medium at 2–8 °C from the time of the procurement to the Eye Bank and, successively, to the operation theatre, with a 14 days maximum storage time. Eusol-C contains Dulbecco’s modified Eagle medium (DMEM), dextran as a deswelling agent, and gentamicin antibiotic. In the control group, 169 human donor corneas were kept in Eusol-C medium at 2–8 °C during transport from the procurement site to the Eye Bank. Here, the tissues were transferred to the organ-culture (OC) medium Tissue-C (AL.CHI.MI.A. S.R.L., Ponte San Nicolò, Italy) within 10 days from procurement, stored at 31 °C for up to 28 days, and then transferred to the deswelling medium Carry-C (AL.CHI.MI.A. S.R.L., Ponte San Nicolò, Italy) at 32 °C for 24 h prior to graft preparation (for DSAEK and DMEK) or shipment at RT to the operation room (PK, DALK). Distribution of tissues to the operating room was performed inside an appropriate isothermal polystyrene container with (Eusol-C group) or without (OC control group) an ice pack. Corneal storage time (time from procurement to surgery) was recorded for each tissue.

### 2.2. Pre-Operative Corneal Evaluation During Storage

All pre-operative evaluations on preserved corneas were performed by an experienced ophthalmologist. Corneal quality parameters were evaluated during storage in Eusol-C (study group) or Tissue-C (OC control group). Donor sclero-corneal tissues were manipulated under a class A laminar flow hood (Bioair Safeflow 1.8, EUROCLONE S.p.A., Pero, Italy).

Slit lamp analysis (CSO, Scandicci, Italy) was performed to evaluate the health status of the corneal epithelium, stroma, and Descemet membrane (DM) [14,15].

The epithelium was classified as: “Normal” (transparent epithelium and absence of defects), “Removed” (absent epithelium), “Exposure epitheliopathy” (presence of iatrogenic epithelial defects), or “Oedematous” (presence of epithelial oedema).

The stroma was graded as follows: “Normal,” indicating the absence of stromal oedema, scars, and foreign bodies; “Oedematous” (presence of stromal oedema); or “Gerontoxon,” indicating the presence of senile changes at the corneal periphery due to lipid infiltration. The “Gerontoxon” category was further divided into three degrees: “Mild,” characterized by gerontoxon affecting one quadrant of the cornea or extending beyond the inner diameter of 10 mm; “Moderate,” defined by gerontoxon affecting two quadrants of the cornea or being confined within the inner diameter of 10 mm; and “Severe,” representing gerontoxon affecting 3 or 4 quadrants of the cornea or being confined within the inner diameter of 10 mm. Corneas exhibiting foreign bodies or scars resulting from previous surgery in the stroma were considered unsuitable for transplantation and were excluded from participation in this study.

DM slit lamp examination categorized corneas with normal DM and corneas with DM folds.

For assessments of the endothelial cell (EC) density (ECD), EC mortality, and EC morphology score, the endothelium was stained with a vital intraocular dye consisting of 1% soluble lutein in combination with 0.04% trypan blue (PhacodyneTM, Alfa Instruments Srl, Casoria, Italy) for 1 min at room temperature (RT) [16]. After removing the excess dye by washing with phosphate-buffered saline (PBS), the tissue was placed in a Petri dish containing 1.4% (*w*/*v* in PBS) sucrose solution for light microscopic evaluation (Nikon Eclipse TE200 bright-field inverted microscope at 100× magnification).

ECD was assessed in the central portion of the endothelium on images of a 100 µm µ 100 ∗m square reticule. At least five counts were made in separate squares, and the mathematical average of the five readings multiplied by 100 gave the ECD estimate, expressed as cells/mm^2^.

EC mortality was assessed by examining nuclei and acellular areas positive for TB staining [14,15]. Observed patterns of mortality (when present) were categorized as follows. If EC mortality was present in all four quadrants, the pattern was considered “Diffuse”. In cases where EC mortality was present on DM folds, the mortality pattern was described as “On folds”.

If areas of mortality were detected peripherally, the categorization used was “Peripheral”. Peripheral EC mortality was further categorized according to the origin of the injury: “Iatrogenic”, “On folds”, “Other”.

EC morphology was assessed, and a score based on a four-grade scale was assigned by an experienced ophthalmologist as previously described [17].

### 2.3. Tissue Eligibility and Preparation for the Surgery

Tissue classification by type of surgery was determined as follows. Corneas were defined as suitable for PK if the absence of epithelial defects, gerontoxon, and other stromal opacities, with detectable intercellular margins and a regular endothelial mosaic, and with ECD ≥ 2300 cells/mm^2^ was observed.

Corneas were considered to have undergone endothelial keratoplasty (DMEK and DSAEK) in the absence of epithelial defects, in the presence of moderate or severe gerontoxon and stromal opacities, with clearly or partially visible intercellular margins and regular or mildly polymorphic endothelial mosaic, as well as with ECD ≥ 2400 cells/mm^2^.

Corneas were defined as eligible for DALK if they had epithelial defects of less than or equal to 50% of the epithelial cells, absent or mild gerontoxon, undetectable endothelial intercellular margins, and irregular endothelial mosaic, with ECD < 2000 cells/mm^2^.

An expert ophthalmologist prepared the lenticule for DMEK and DSAEK at the Eye Bank of Rome under a laminar flow hood. Lenticules for DALK were prepared in the operating room immediately prior to transplantation.

The DMEK grafts were prepared after staining with a solution containing trypan blue 0.05% *w/v* (RS-Blue; AL.CHI.MI.A. S.R.L., Ponte San Nicolò, Italy) for 2 min at RT to improve visualization of the endothelium-Descemet’s membrane (EDM) complex. After washing with PBS, the EDM complex was peeled away from the stroma using the Scuba technique (with endothelium covered by Carry-C liquid), leaving a peripheral hinge. The EDM complex was then repositioned back on the stroma with sterile swabs, and the cornea pre-stripped was sent to the operating theatre in Eusol-C (study group) or Carry-C (OC control group), ready to be punched at the desired diameter.

DSAEK graft preparation was performed using the Evolution 3E Console Control Unit (Moria SA, Anthony, France). The pressure in the artificial anterior chamber (AAC) was controlled by the ACP system (Moria SA, Anthony, France) and set at 200 mmHg. Tone was maintained using sterile Carry-C in both experimental groups, which was injected into the AAC circuit with a syringe. The cut was performed using the One Use Plus microkeratome (Moria SA, Anthony, France). The selection of appropriate microkeratome head sizes based on central corneal thickness (CCT) allowed the achievement of a target corneal graft thickness between 70 and 150 μm, which was measured using the anterior segment Optical Coherence Tomography (Visante AS-OCT, Carl Zeiss Meditec, Germany; CSO AS-OCT, Opto Medica Oftalmologia Srl, Rome, Italy), with tissue supported in a viewing chamber. DALK grafts were prepared by manually peeling off the DM-endothelium complex from the underlying corneal stroma.

### 2.4. Surgical Techniques

A single experienced surgeon (AP) performed all surgeries, and all procedures did not differ significantly from current standards [18,19,20,21]. Specifically, in the DALK technique, the trephination size ranged from 8.00 mm to 8.50 mm (Moria Surgical, Antony, France), and the intended depth of trephination was set to 90% of the lowest pachymetric value measured at the trephination site by AS-OCT [22]. In addition, anterior keratectomy was performed in all subjects before attempting air injection. PK was performed when intraoperative extensive DM perforation precluded lamellar keratoplasty. For DALK, the big-bubble technique was attempted in all cases. In cases where a big bubble could not be achieved, predescemetic DALK was performed using a crescent knife to remove as much of the corneal stroma as possible.

Grafts were oversized by 0.25 mm and sutured to the recipient beds using 16 interrupted 10/0 nylon sutures or a 16-bite continuous suture combined with 8 interrupted 10/0 nylon sutures. The surgeon used the same suture tension for both keratoplasty techniques. Suture tension regulation was performed intraoperatively with a Maloney handheld keratoscope (Bausch + Lomb Storz Ophthalmic Instruments, Vaughan, Canada).

For endothelial keratoplasty, the donor tissue was inserted through a corneal incision of 4.1 mm and 2.8–3.0 mm for DSAEK and DMEK, respectively. The size of the descemetorhexis was approximately 8.5–9.5 mm in both procedures. An intracameral air bubble or gas (20% sulfur hexafluoride; GOT Multi SF6, AL.CHI.MI.A. Srl) was used to facilitate tamponade of the graft to the host cornea.

### 2.5. Follow-Up Evaluations

The incidence of complications in all surgeries (i.e., primary graft failure and postoperative infection) was evaluated on the day after surgery and during the entire follow-up. Also, re-bubbling rates in DMEK and DSAEK interventions were recorded.

The Corrected Distance Visual Acuity (CDVA) was examined at three (Eusol-C group: n = 10; OC control group: n = 69) and six (Eusol-C group: n = 26; OC control group: n = 74) months with the Snellen decimal system after surgery.

Corneal transparency was evaluated by one surgeon (AP) using slit lamp analysis (CSO, Opto Medica Oftalmologia Srl, Italy) at 24 h (Eusol-C group: n = 92), 3 months (Eusol-C group: n = 15; OC control group: n = 70), and 6 months (Eusol-C group: n = 27; OC control group: n = 74) post-transplantation. Corneal transparency was categorized as follows: “Transparent,” indicating complete clearness of the cornea; “Moderate,” indicating the presence of partial oedema distributed in discrete corneal areas; “Oedematous,” indicating the presence of complete oedema; additionally, the presence of neovascolarization or ulcer was reported. Potential evidence of infection was also evaluated during slit-lamp analysis. Anterior segment OCT (CSO AS-OCT, Opto Medica Oftalmologia Srl, Italy) was used to assess the adherence of DMEK and DSAEK grafts to the recipient stroma. Graft failure was defined as irreversible graft stromal oedema and corneal opacity according to Borderie et al. [23]. Similarly, graft rejection was defined as any new corneal oedema or haze depending on the site primarily affected by the reactive immunologic process [23,24].

### 2.6. Sample Size Estimation and Statistical Analysis

For sample size estimation, a power analysis was performed using Sealed Envelope™ software (version 30.1.0) available online on CDVA data obtained at 6 months follow-up. The minimum sample size to demonstrate equivalence with a continuous outcome trial between the two corneal preservation techniques was estimated to be 22 patients per group, according to an accepted difference among the two study groups of 2 CDVA units, a standard deviation (SD) of 2 CDVA units, an alpha level of 0.05, and a power of 90%.

Statistical analysis was performed using Excel Microsoft Office 2019 (Microsoft Corp., Redmond, WA, USA) software provided with the plugin Real Statistics Resource Pack (https://real-statistics.com/). Normality of data distribution was evaluated using the Shapiro–Wilk test. Intra-group and inter-groups comparisons of CDVA were compared using the two-tailed Mann–Whitney Test for Two Independent Samples. Statistical analysis of the distribution of corneal transparency at various follow-up time points was performed using Pearson’s chi-squared test (with contingency tables 3 × 3) according to Shan & Gerstenberer [25] using an online calculator (https://www.socscistatistics.com/tests/chisquare2/default2.aspx). Differences yielding *p* < 0.05 were considered statistically significant.

## 3. Results

### 3.1. Pre-Operative Examination of Corneal Quality Parameters

Table 1A describes the donor data of the transplanted corneas belonging to the experimental (Eusol-C) group and the OC control group. The mean donor age was 65 ± 12 years in both groups, with a higher percentage of male donors (Eusol-C: 59.8%; OC control: 60.9%) compared to female donors. The mean death-to-preservation time was 9.6 ± 7.7 and 10.5 ± 7.2 h for the Eusol-C and OC control groups, respectively.

Table 1B presents the pre-operative quality parameters of donor corneas. Examination was performed on average at 3.1 ± 1.5 and 19.2 ± 5.3 days after procurement for the Eusol-C and OC control groups, respectively.

The average hypothermic storage duration in the Eusol-C group was 7.7 ± 2.5 days. In the OC control group, corneas were maintained in Eusol-C at 2–8 °C for an average of 5.0 ± 3.3 days prior to transfer to Tissue-C and corneal organ-culture at 31 °C for an average of 14.2 ± 4.0 days.

The ECD was measured at 2398 ± 354 cells/mm^2^ for the Eusol-C group and 2256 ± 328 cells/mm^2^ for the OC control group, and the average EC morphology score was 3.4 ± 0.7 and 3.5 ± 0.5 (on a scale of 0 to 4) for the Eusol-C and OC control groups, respectively. Approximately 26% of corneas in the Eusol-C group exhibited no endothelial cell mortality, while the remaining tissues displayed mortality primarily on the periphery (34%) and in the folds (19%). Conversely, the OC control group showed a different distribution of endothelial cell mortality, with approximately 42% of corneas exhibiting no endothelial cell mortality, a lower percentage (15%) exhibiting mortality on the periphery, and a higher percentage of corneas showing mortality in the folds (40%).

**Figure 1:**
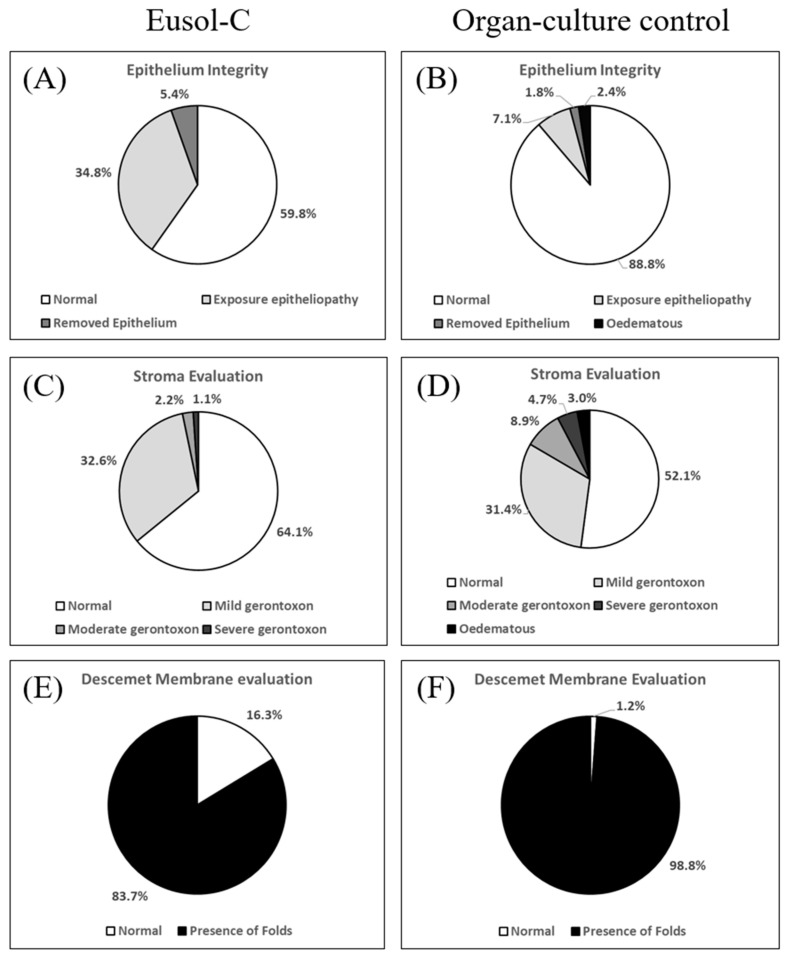
Pre-operative slit lamp evaluations of human corneas during storage in Eusol-C medium at 2–8 °C (**A**,**C**,**E**) and OC control (Tissue-C) at 31 °C (**B**,**D**,**F**). (**A**,**B**) evaluation of epithelium integrity; (**C**,**D**) assessment of gerontoxon occurrence in the stroma; (**E**,**F**) assessment of presence of folds in the Descemet membrane.

Figure 1 illustrates the categorization distributions for epithelial integrity (Figure 1A,B), stroma condition (Figure 1C,D), and DM condition (Figure 1E,F) evaluated with a slit lamp on the same day of light microscopy analysis. Most corneas exhibited a normal epithelium (Eusol-C: 60%; OC control: 89%), followed by corneas with exposure epitheliopathy (Eusol-C: 35%; OC control: 8%). A significant proportion of the stromas appeared normal (Eusol-C: 64%; OC control: 52%), with a smaller percentage exhibiting mild gerontoxon (Eusol-C: 33%; OC control: 31%). The predominant condition observed in the DM was the presence of folds (Eusol-C: 84%; OC control: 99%).

### 3.2. Surgery

**Figure 2:**
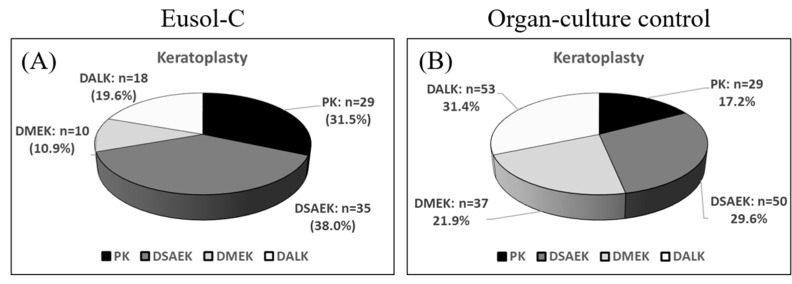
Proportion of cornea transplantation surgeries of corneas stored in Eusol-C medium at 2–8 °C (**A**) and OC control (Tissue-C) at 31 °C (**B**). PK: Penetrating Keratoplasty; DALK: Deep Anterior Lamellar Keratoplasty; DMEK: Descemet Membrane Endothelial Keratoplasty; DSAEK: Descemet Stripping Automated Endothelial Keratoplasty.

Figure 2 shows the proportions of different surgeries performed in the study. DSAEK was the most common surgical procedure in the Eusol-C group (38%), followed by PK (32%), DALK (20%), and DMEK (11%), while DALK was the most performed intervention in the OC control group (31%), followed by DSAEK (30%), DMEK (22%), and PK (17%). The corneas designated for DSAEK surgery exhibited an average post-cut graft thickness of 113 ± 21 µm and 103 ± 21 µm in the Eusol-C group and the OC control group, respectively.

There was no re-bubbling for all endothelial keratoplasty surgeries (DMEK + DSAEK) in both groups (n = 45 total interventions in the Eusol-C Group and n = 87 total interventions in the OC control group).

No intraoperative complications were documented across all surgeries performed.

### 3.3. Post-Operative Follow-Up

Table 2 shows the postoperative follow-up parameters examined after cornea transplantations.

At 24 h surgery, 81.3% of patients who received donor corneas stored in hypothermic Eusol-C medium had transparent corneas, 16.5% had partially transparent corneas, and 2.2% had oedematous corneas. The percentage of transparent corneas progressively increased at three (86.7%) and six months (92.6%), with no statistical significance observed across the time points (Pearson’s chi-squared test: *p* = 0.2462).

The percentage of transparent transplanted corneas from the OC control group was 94.3% at three months and 94.6% at six months, with the difference that was not statistically significant (Pearson’s chi-squared test: *p* = 0.6486).

No statistical differences (Pearson’s chi-squared test) were observed in corneal transparency comparing Eusol-C and OC control groups at three (*p* = 0.2795) and six (*p* = 0.7064) months follow-up.

Mean CDVAs of transplanted corneas from the Eusol-C group at three and six months were, respectively, 4.5 ± 4.0/10 and 5.4 ± 3.7/10, and the difference was not statistically significant (two-tailed Mann–Whitney Test for Two Independent Samples: *p* = 0.5898).

Transplanted corneas from the OC control group had mean CDVAs of 5.0 ± 2.9/10 and 5.7 ± 2.9/10 at the three- and six-months follow-up, respectively, and the difference was not statistically significant (two-tailed Mann–Whitney Test for Two Independent Samples: *p* = 0.1473).

No statistical differences (Mann–Whitney Test for Two Independent Samples) were observed among the two groups at three (*p* = 0.6207) and six (*p* = 0.9538) months CDVA follow-up.

No cases of graft failure were observed for up to 3 months in both groups.

One case (1.1%) of graft failure secondary to immunological rejection was recorded in one transplanted cornea belonging to the Eusol-C group at the 6-month follow-up after a DSAEK surgery, presenting corneal oedema and keratic precipitates. The patient did not respond to anti-rejection therapy, and the patient underwent a second transplantation.

In the OC control group, two cases (1.2%) of corneal rejection were recorded at the six-month follow-up: one case after DMEK surgery, with inferior oedema and endothelial precipitates, and one case after DALK surgery, with oedema and deep folds. Both patients underwent a second transplantation.

## 4. Discussion

This retrospective study evaluated the clinical outcomes of various keratoplasty procedures using two different methods of preserving human donor corneas. The experimental group consisted of 92 corneas stored in Eusol-C medium at 2–8 °C, with an average storage duration of 7.7 days in this study. This was compared to a control group, consisting of 169 transplanted corneas stored in the most commonly adopted storage technique in Europe, i.e., organ-culture at 31 °C in Tissue-C medium for up to 28 days, with an average duration of 14.2 days in this study.

The results presented confirm the efficacy and safety of Eusol-C as a hypothermic preservation medium intended for human corneas and provide insights into its potential impact on corneal transplantation outcomes. Indeed, regardless of the surgical procedures used (PK, DALK, DSAEK, and DMEK), no adverse events were reported the day after surgery, demonstrating the versatility and applicability of Eusol-C medium for the preservation of corneas subjected to different keratoplasty techniques. Moreover, no significant issues were observed during DM peeling for DMEK graft preparation, and an accurate selection of the microkeratome head based on corneal thickness allowed for an average graft thickness of 113 µm without corneal perforation events during DSAEK graft preparation. In addition, a 0% rebubbling rate was observed after DMEK and DSAEK in both the Eusol-C and OC control groups. Noteworthy, grafts undergoing rebubbling have a significantly higher rate of endothelial cell loss than those not undergoing rebubbling [26].

Regarding the postoperative assessments, the results showed a considerable proportion (81.3%) of transparent grafts from corneas in the Eusol-C group during the 24 h observation period, indicating a favorable initial surgical outcome. Furthermore, this observation supports that the transplanted donor graft promptly resumes its physiological function within both recipients ocular tissues, including the restoration of endothelial sodium-potassium pump (Na+/K+-ATPase) activity, which is a crucial requirement for maintaining corneal transparency [27,28]. In addition, the percentage of transparent corneas in the Eusol-C group increased at three months, and the mean CDVA was 4.5/10, indicating favorable visual recovery. Similarly, at the 6-month evaluation, most grafts (25 out of 27: 92.6%) continued to show corneal transparency, and the mean CDVA increased to 5.4/10.

Furthermore, no graft failures were reported at the 3-month follow-up either in the Eusol-C or OC control groups, confirming the overall success of the surgeries and the stability of the transplanted corneas.

We report a case of graft failure due to endothelial rejection six months after DSAEK of a cornea stored in hypothermic storage, in which the patient underwent a second transplantation.

It is noteworthy that the contralateral cornea from the same donor was used for another DSAEK procedure, with almost equivalent storage time and pre-operative evaluation quality parameters, and it was successfully transplanted into another recipient. This observation suggests that the endothelial failure observed in this study was most likely due to the recipient’s immune response rather than the inadequate quality of the transplanted cornea [29]. Remarkably, one graft rejection out of 92 transplantations (1.1%) at the 6-month follow-up is consistent with what was observed in the OC control group (two graft rejections out of 169 transplantations: 1.2%) as well as with previous literature reporting rejection rates in corneal transplantation [29,30,31,32,33,34].

This study has some limitations. First, the analysis on hypothermic storage medium Eusol-C is limited by a relatively modest sample size (n = 92) of corneas. This limitation is mainly due to the prevailing practice within European eye banks, where hypothermic storage (2–8 °C) remains relatively uncommon, with organ-culture (31–37 °C) being the predominant method [5,6,14]. Among the corneas obtained from the Eye Bank of Rome during the study period, approximately 10% were designated for hypothermic storage, limiting the sample size of this study. Furthermore, the small sample size might have contributed to the lack of statistically significant differences observed among the different follow-up time points of the Eusol-C group regarding CDVA and corneal transparency.

Another limitation of this study is that a relatively short follow-up may not adequately encompass long-term complications or outcomes. Therefore, future prospective studies with larger sample sizes and longer follow-up periods are warranted to validate these findings and to explore the long-term effects of Eusol-C storage.

On the other hand, this study provides valuable insights into the clinical comparison of the two almost universally used techniques for corneal storage: hypothermic storage and organ-culture. As shown by the results presented here, these methods differ significantly in both technical and logistical aspects. In this study, hypothermic storage in Eusol-C allowed a shorter preservation time than the OC control group, reflecting the distinct characteristics and timeframes of the two preservation methods. Noteworthy, several reports [8,35,36] have shown that EC mortality is directly proportional to the length of hypothermic storage time, indicating that the duration of cold storage should be minimized whenever possible. Another aspect to consider is that corneal cells stored in hypothermic storage are metabolically dormant, while cell metabolism is active in organ-cultured corneas. Indeed, corneas in the OC control group showed superior epithelial integrity in our study, consistent with previous findings [37]. Also, the presence of a deswelling agent in hypothermic storage media helps maintain physiological thickness, while in organ culture, corneas tend to swell [5,6]. Swelling contributed to the appearance of epithelial and stromal oedema and the occurrence of DM folds in almost all (99%) of the organ-cultured corneas considered in our study, where EC mortality occurred, as previously reported [38,39].

Despite the above-mentioned differences, both preserving methods have been demonstrated to be safe and effective in preserving corneal quality within their defined timeframes [5,6,36]. Accordingly, the evaluated clinical outcomes (CDVA and corneal transparency) did not show statistically significant differences between patients in the Eusol-C group and those in the OC control group at three- and six-month follow-up. This indicates equivalent functional outcomes of corneal transplantation from grafts preserved with either of these two preservation techniques, despite their technical differences.

## 5. Conclusions

In summary, the data collected support the use of Eusol-C as a practical hypothermic storage medium for donor corneas. The favorable clinical results, including advantageous graft preparation within the eye bank, remarkable rates of graft transparency, and good CDVA, collectively highlight the safety and reliability of Eusol-C as a hypothermic preservation solution for human donor corneas intended for transplantation using various keratoplasty techniques.

## Figures and Tables

**Table 1:** (**A**) Demographic donor cornea data and (**B**) measured pre-operative cornea quality parameters (means ± SDs) of tissues stored in Eusol-C at 2–8 °C (left column) or OC control (Tissue-C) at 31 °C (right panel). EC: Endothelial cells. ECD: Endothelial Cell Density. * EC mortality was evaluated on n = 85 corneas in the Eusol-C group. # Corneas may exhibit multiple patterns of EC mortality and thus can belong to two different categories at the same time.

	Eusol-C (n = 92)	OC Control (n = 169)
**(A) Donor data**		
Donor Age (years)	65 ± 12 Range: 24–81 Median: 67	65 ± 12 Range: 6–80 Median: 69
Donor sex	Female: n = 37 (40.2%) Male: n = 55 (59.8%)	Female: n = 66 (39.1%) Male: n = 103 (60.9%)
Death-to-preservation time (h)	9.6 ± 7.7	10.5 ± 7.2
**(B) Pre-operative cornea quality parameters**		
Corneal storage time (days)	7.7 ± 2.5	5.0 ± 3.3 in Eusol-C from procurement to the Eye Bank; 14.2 ± 4.0 Organ-Culture in Tissue-C.
Evaluation time point (days from procurement)	3.1 ± 1.5	19.2 ± 5.3
ECD (cells/mm^2^)	2398 ± 354	2256 ± 328
EC Morphology (score) [17]	3.4 ± 0.7/4	3.5± 0.5/4
EC Mortality *^,#^	Absent: n = 22 (25.9%)	Absent: n = 71 (42.0%)
	Diffused: n = 15 (17.6%)	Diffused: n = 26 (15.4%)
	On Folds: n = 16 (18.8%)	On Folds: n = 68 (40.2%)
	Peripheral–Iatrogenic: n = 25 (29.4%) Peripheral–Other: n = 4 (4.7%)	Peripheral–Iatrogenic: n = 3 (1.8%) Peripheral–On Folds: n = 10 (5.9%) Peripheral–Other: n = 13 (7.7%)

**Table 2:** Post-operative follow-up of corneal transparency at 24 h, 3 and 6 months, and corrected distance visual acuity (CDVA) and graft failure/rejection at 3 and 6 months of Eusol-C and OC control groups.

FOLLOW-UP		Eusol-C			OC Control	
		24 h (n = 92)	3 Months (n = 15)	6 Months (n = 28)	3 Months (n = 70)	6 Months (n = 74)
Corneal Transparency	Transparent	74 (81.3%)	13 (86.7%)	25 (92.6%)	66 (94.3%)	70 (94.6%)
	Moderate Oedema	15 (16.5%)	2 (13.3%)	0 (0.0%)	1 (1.4%)	1 (1.4%)
	Oedematous	2 (2.2%)	0 (0.0%)	2 (7.4%)	1 (1.4%)	3 (4.1%)
	Other	0 (0.0%)	0 (0.0%)	0 (0.0%)	New Vessels formation: 1 (1.4%) -Ulcer: 1 (1.4%)	0 (0.0%)
CDVA		/	4.5 ± 4.0/10 (n = 10)	5.4 ± 3.7/10 (n = 26)	5.0 ± 2.9/10 (n = 69)	5.7 ± 2.9/10 (n = 74)
Graft failure/rejection		/	No graft failure detected	n = 1 case (1.1%): -endothelial rejection after DSAEK surgery, with oedematous cornea	No graft failure detected	n = 2 cases (1.2%): -endothelial precipitates after DMEK -rejection with deep folds after DALK

## Data Availability

Data available upon reasonable request.
